# Rural Tourism and the Sustainable Development Goals. A Study of the Variables That Most Influence the Behavior of the Tourist

**DOI:** 10.3389/fpsyg.2021.722973

**Published:** 2021-07-23

**Authors:** José María López-Sanz, Azucena Penelas-Leguía, Pablo Gutiérrez-Rodríguez, Pedro Cuesta-Valiño

**Affiliations:** ^1^Economics and Business Management Department, Faculty of Economics, Business and Tourism, Universidad de Alcalá, Alcalá de Henares, Spain; ^2^Department of Business Administration, Faculty of Economic and Management Sciences, Universidad de León, León, Spain

**Keywords:** motivation, destination imagen, satisfaction, rural tourism, SDG

## Abstract

Tourism is an activity that contributes directly and indirectly to the development of rural areas. But this development needs to be sustainable. To do this, appropriate policies that positively influence these areas from an economic, social and cultural point of view must be implemented. All this in accordance with the Sustainable Development Goals. This study will analyze the contribution of rural tourism to develop and implement policies to promote sustainable tourism that creates jobs and promotes local culture and products. The variables that most influence the tourist behavior, motivation, the destination image, and the satisfaction obtained by the tourist will be analyzed. After an exhaustive review of the literature, an empirical investigation was carried out with 1,658 valid surveys among rural tourists in Soria, a Spanish province with one of the highest levels of depopulation. A structural equation model was drawn up to discover the relationships between the variables. The results demonstrated the importance of the motivation in the formation of the destination image, as well as satisfaction with the trip. In the same way, we will verify which component of the image of the destination (affective or cognitive) has the most influence on their formation, and how the image of the destination, like motivation, influences tourist satisfaction. The proposed model could be used in many studies that analyze the different variables that influence consumer behavior since its reliability and predictive capacity have been proven. The results of the study can also be used by the authorities to design or modify the most appropriate strategies that influence rural tourism, specially promoting the destination image as a variable that positively influences tourist satisfaction.

## Introduction

This study is an original investigation of the rural tourists' behavior, attending to the most important variables that help to understand this behavior. It is analyzed how policies focused on rural tourism should be in line with Sustainable Development Goals defined by the UN in 2017, especially with objective 8 “Decent Work and Economic Growth,” to promote sustainable tourism, which creates jobs and promotes culture and local products, as can be seen in the goal 8.9 of that goal number 8.

Rural tourism has gained broad acceptance in Spain. The wide range of accommodation and activities included in the definition of “Rural Tourism” makes it a very attractive option to consumers. In Spain, it is now an important alternative to sun and beach tourism, which has traditionally been a very popular choice of vacationers.

As a consequence, for depopulated and depressed areas in Spain, this kind of tourism has become an additional economic activity, so they no longer depend exclusively on primary activities such as agriculture and livestock. There are extensive opportunities for agrotourism, combining tourism with agriculture-related activities, which indicate the potential synergies between them. The local authorities managing rural tourism must therefore implement policies to promote its development. For Polo (2010), the development of the rural tourist activity is very suitable for improving the development of the rural areas, likewise Marzo-Navarro (2017) stated that rural tourism promotes the development and economic growth of the destination areas, for which it is a priority to achieve the objectives of economic, sociocultural, and environmental sustainability. The World Tourism Organization (UNWTO) (2021) has recognized that “tourism is one of the driving forces of global economic growth and is currently responsible for the creation of 1 in 11 jobs. By giving access to decent work opportunities in the tourism sector, society, in particular, young people and women, can benefit from improved skills and professional development. The sector's contribution to job creation is recognized in target 8.9: by 2030, devise and implement policies to promote sustainable tourism that creates jobs and promotes local culture and products.” To this end, it is thus very important to analyze a range of variables and components that may influence rural tourism behavior.

Among the most influential variables, satisfaction is a key factor that indicates what the trip has meant to the tourist. Many studies have demonstrated the importance of perceived value and satisfaction in tourist behavior (Barsky and Labagh, 1992; Tam, 2000; Choi and Chu, 2001; Tian-Cole and Cromption, 2003; Petrick, 2004; Yoon and Uysal, 2005; Hutchinson et al., 2009; Kim et al., 2009; Jin et al., 2013; Asgarnezhad et al., 2018; Chin et al., 2019; Penelas-Leguía et al., 2019; Castro et al., 2020). Several studies considered “word-of-mouth” a very important factor to explain the future behavior and it is the link between satisfaction and loyalty (Hutchinson et al., 2009; Kim et al., 2009; García, 2011; Lai et al., 2018; Xu et al., 2020). It is, however, essential to discover how the tourist's image of their destination, and their other motivations, drive them to choose that destination. To Tasci and Gartner (2007), destination image is a key factor in successful tourism development. To Ejarque (2016), this image has a vital importance in tourists' selection processes. And a tourist's motivation has an important impact on destination image formation, as Li et al. (2010) and Sancho and Álvarez (2010) explained in their studies. It is, however, interesting to investigate the influence motivation has on overall visitor satisfaction, as per Albayrak and Caber (2018).

## Conceptual Framework and Hypothesis

### Research Framework

**Figure 1:**
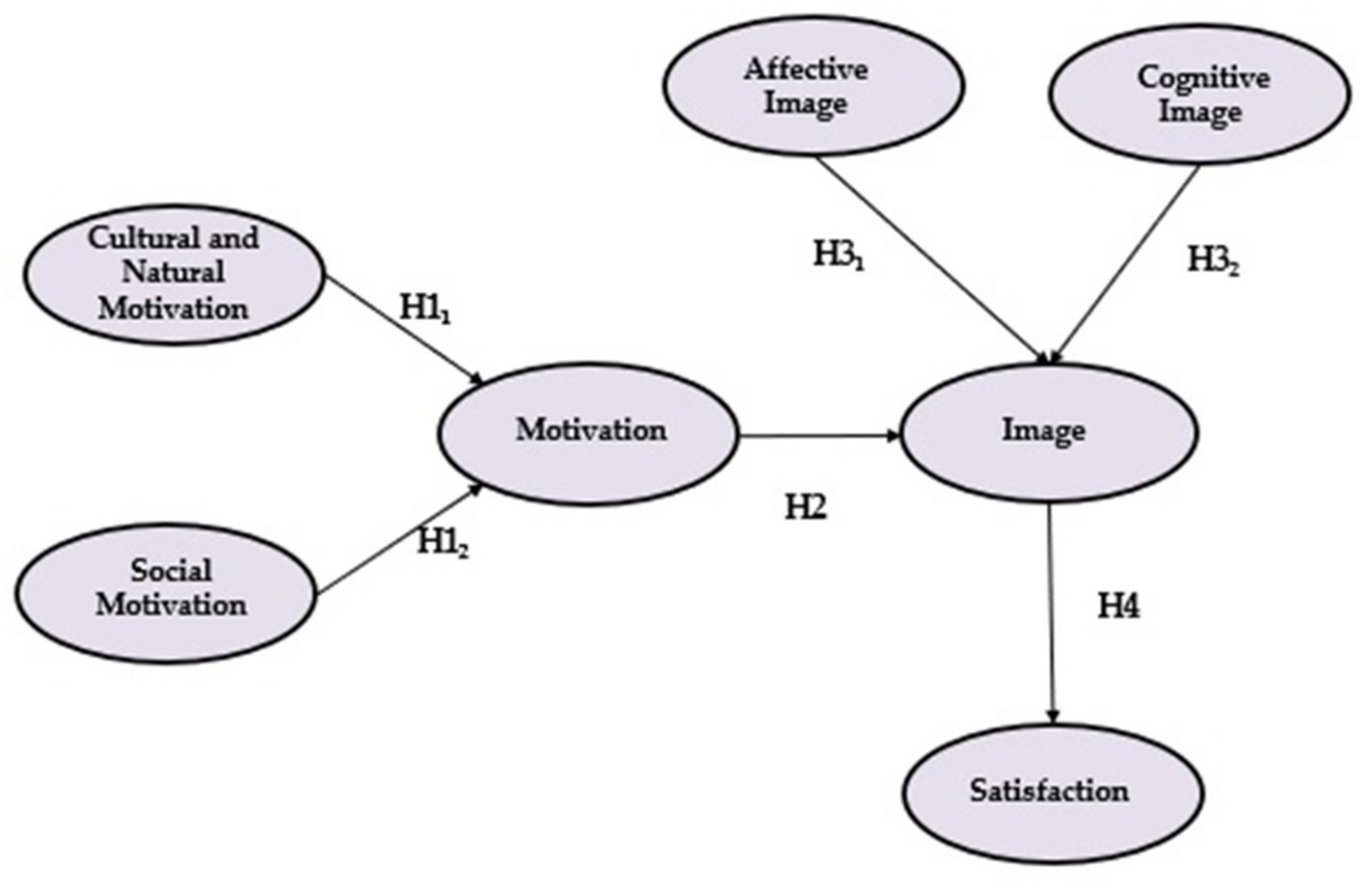
Proposed theorical model.

In this research, we reviewed the literature on the variables that affect tourist behavior (motivation, image and satisfaction). We then used the results of this review to lay the foundations of a behavior model using Structural Equations, with Partial Least Squares (PLS) as the chosen method, as you can see in Figure 1. This will indicate the links between those variables and the strength of these relationships. Thus, the main objective of the research is to analyze the links between tourist motivation, destination image and vacation satisfaction. And as secondary objectives, which complement the analysis, we expose:

- To research how motivations influence destination image formation.
- To analyze the link between destination image and satisfaction with rural tourism
- To research the importance of the affective and cognitive components of the image in forming the overall destination image.

### Research Hypothesis

Motivation has been widely studied by various authors and in different areas, from psychology to sociology and marketing. Motivation is the driving force of the process. A consumer can have a positive attitude to the purchasing process, an excellent image of the product or service, but if they aren't strongly motivated, the process doesn't begin. A motive, as Santesmases (2012, p. 261) explains, is “the reason why the consumer purchases the product.” Consumers buy something because they get a benefit, and those benefits satisfy needs. Motivation is therefore, according to Santesmases (p. 261), “a general disposition that leads to the behavior aimed at obtaining what the consumer wants.” Kotler (2016, p. 199) defines motive as “a need that is sufficiently pressing to drive the person to act.”

From the tourism point of view, motivation is one of the most important and most extensively studied variables. Wong et al. (2018), point out the influence that motivations have on the tourism process, especially on the tourist. One of the early studies was by Dann (1977). He attempted to explain the reasons why people travel, as well as their choice of destination. This was the first-time push and pull factors were discussed.

One of the most relevant and important studies of this topic is by Crompton (1979). This author found nine key motives for a tourist's choice, seven of which were categorized as socio-psychological (escape from a perceived mundane environment, exploration and evaluation of self, relaxation, prestige, regression, enhancement of kinship relationships and facilitation of social interaction), and two cultural motives (novelty and education). The socio-psychological motive, also referred to as “push” motives, explain the wish to take holidays, while cultural motives, also called “pull” motives, explains the choice of the destination or the kind of destination. In addition to this author, Crandall (1980), based on Crompton's work, continues the explanation of the value of motivation in tourist behavior, and list seventeen personal motives. These are, clearly, an extension to Crompton's nine motives.

Other authors, such as Line et al. (2018), focus on the importance of motivations in tourist behavior. They explain the importance of motivation, with a special link between motivation and sustainability programs. González and Vallejo (2021), they also explained that importance. Polo et al. (2016) evaluate the motivations with influence in the rural tourist in Spain, their behavior and the different strategies, and Prebensen et al. (2010) study tourist behavior, in this case, the sun and sand tourist and the link between motivations to travel, tourist satisfaction and intentions to communicate with others by word-of-mouth.

Regarding the practice of rural tourism, Penelas-Leguía et al. (2019) classified the different motivational factors into which tourist motivation is divided. These factors were natural and cultural motivations, social motivations, personal motivations, novelty motivations and escape motivations, reaching the conclusion that natural and cultural and social motivations are the ones that have the most influence on the formation of tourist motivation. Buffa (2015), also focused on the study of cultural and natural motivations in rural tourism practice, concluding that tourists, especially the youngest, feel motivated when traveling to discover new cultures, new natural spaces, contemplate the natural and artistic heritage, be in contact with the local population and contact with nature. Han et al. (2017), continues in this line, on the importance of nature and natural heritage in tourism decision-making. Luo and Deng (2007), exposed the environment and nature as one of the main reasons that move tourists to visit a tourist area, while Gnoth and Zins (2010) and Kim and Prideaux (2005), considered that motivation cultural and knowing the cultural heritage of the area, were the main reason that moves the tourist. Regarding social motivations, several studies point out this type as the main factor when making decisions by tourists. Van der Merwe et al. (2011) exposed the great importance of these motivations, after an exhaustive review of the literature. Lee et al. (2004) and Park et al. (2008), focused their studies on the key importance of social motivations in the tourist's behavior. Moreno et al. (2012), exposed the three main types of motivations that move tourists, highlighting cultural motivations and social motivations, as well as those of “self-expression.”

Therefore, we observe the importance of natural and cultural and social motivations in tourist decision-making, so we propose the following hypotheses:

Hypothesis 1.1 (H11): “Cultural and natural motivation is the main dimension of the tourist motivation.”
Hypothesis 1.2 (H12): “Social motivation has an important relevance in the formation of tourist motivation.”

About the link between tourist motivation and destination image, several authors have studied this influence. For Li et al. (2010), destination image is an essential component of tourist destination success, because if the place has a recognizable image, it will be more likely to be chosen by tourists as a place for recreation and leisure. In this study, they recognize three motivational factors: intellectual, belonging and escape. Each of them has a direct effect on the cognitive component of the image, but for the escape dimension of the motivation, this effect is negative. For the affective component of the image, the relationship is direct if we focus on the escape motivation as well as on the cognitive component.

Sancho and Álvarez (2010) point out the importance of motivation in the decision-making process of going on a trip and determining where to go. They consider five main motivations: past experiences, physical, cultural, interpersonal, social and prestige. They concluded that interpersonal and social motivations have a direct effect on the cognitive component of the image and on the overall image. They also found that the cognitive component has a direct effect on the affective component, which in turn affects the overall image. Madden et al. (2016) also analyzed this link, carrying out an exhaustive analysis of the literature, as did Dagustani et al. (2018), Pereira and Hussain (2019) and Santoso (2019), who presented a behavior model studying the relationship between motivations, destination image and tourist satisfaction. In addition to these authors, many others have studied the close relationship between motivations and destination image, and we would highlight the studies by Mayo and Jarvis (1981), Michie (1986) and Gong and Sun Tung (2017). It is also worth highlighting the study of Hwang et al. (2020), who study the relationship between the destination image and the tourist motivations, but inversely, how the destination image influences the formation of the tourist motivations.

We therefore conclude there is an important link between tourist motivations and destination image formation. Thus, we define the following as hypothesis:

Hypothesis 2 (H2): “Tourist motivation significantly positive influences destination image formation.”

Image is a key factor when tourists are choosing their destinations, and crucial when planning a trip (Marine-Roig and Ferrer-Rosell, 2018). As Beerli and Martín (2002) point out, the image has an important impact on tourist behavior, and varies from person to person. In the same way, Foroudi et al. (2018, p. 97) explain that “a positive image is much more likely to be taken into consideration and probably chosen in the decision process.” But this image has to be protected, because it can turn into a negative variable, as Bachiller et al. (2005) explain when they state the problem that overcrowding causes in the destination image. Additionally, Alrawadieh et al. (2019), point out that this feeling of overcrowding doesn't influence the image, but does influence intentions to visit the place again.

What does “destination image” mean? Many authors have contributed their own definitions. To Crompton (1979), destination image is “the sum of all beliefs, ideas and impressions that people associate with a destination.” In 1993, Echtner and Ritchie (1993, p. 3) defined it as “perceptions of individual destination attributes, as well as, total, holistic impressions.” Baloglu and McCleary (1999, p. 870) considered destination image to be “an individual's mental representation of knowledge (beliefs), feelings and global impression about an object or destination.” Sanz (2008), p. 98 explains to us that destination image is “the global perception of a destination, in other words, the representation in the tourist's mind of what he or she feels and knows about it.” And López-Sanz et al. (2021b) defined destination image as the overall mental impressions each person has of a place or destination formed by knowledge as well as by the feelings the destination produces in them.

All these definitions have a common link. Destination image is made up of two components: the cognitive and affective components. Baloglu and McCleary (1999, p. 870) defined both. For them, the cognitive component “refers to beliefs or knowledge about a destination's attributes,” whereas the affective component “refers to feelings about a destination, or attachment to it.” Many other authors, however, have written about the difference between the cognitive and affective components. Beerli et al. (2003) explain that the affective component is “individuals' feelings toward a destination or as an emotional response of individuals to a place,” while the cognitive component “is knowledge about a destination.” To Lee et al. (2008, p. 814), the cognitive component “derives from factual information,” while the affective component “can be viewed as one's diffuse feelings about a specific tourism destination.” Other authors, such as Zhang and Zhang (2020), emphasize this division of destination image. We can therefore state that destination image is formed by the link between two components: cognitive, related to beliefs and knowledge acquired from external information sources or experience; and the affective component, related to feelings. These are strongly linked, in such a way if that the cognitive component changes after the first vacation, the affective response will also be affected. The overall image is made up of these two components. A destination choice depends on the overall image, and when we refer to destination image, we mean the overall image.

We have analyzed the components into which the overall destination image is divided. It is now necessary to focus on the elements that influence the tourist in forming that image. Several authors have discussed these variables. For Baloglu and McCleary (1999), the variety and type of information sources, and the tourist's age, education and motivation all influence destination image formation. For Beerli and Martín (2002), the perceived image of a place is formed by the interaction of several factors, such as the tourist's motivations, previous experience, preferences and other personal characteristics (sex, age, etc.); other psychological factors such as values, personality, lifestyle, etc. also have an influence. To Sirakaya et al. (2001), consumers' choice processes are influenced by their motivations, attitudes, beliefs and values, as well as other types of factors, such as time. Gunn (1993) states that destination image undergoes a constant process of modification. For this author, there are several steps in image formation. First of all, a destination image is generated from previous information (documentaries, acquaintances' experiences, etc.). Later, due to promotional information such as brochures, an induced image is generated. Nowadays, for those referred to as “2.0 tourists,” the importance of “on-line reputation” is increasing, so innovation is essential to building an initial image of destinations, especially the more traditional ones. For some places, destination image may be reinforced by heritage-related cultural events that are publicized over social networks (Campillo-Alhama and Martínez-Sala, 2019). This image may help individuals choose a destination, depending on their motivations. After the vacation and the tourist's personal experience, a final image is generated. For Um and Crompton (1990) and Ugarte (2007), the perceived image of a place will be formed by the interaction of the projected image (the destination image the promotional information projects) and the individual's needs, motivations, experience, preferences and personal characteristics, and this image is very resistant to change, even in times of economic crisis (Gkritzali et al., 2018). Thus, we propose the following hypotheses:

Hypothesis 3.1 (H31): “Affective destination image has a positive influence on destination image formation.”
Hypothesis 3.2 (H32): “Cognitive destination image has a positive influence on destination image formation.”

Overall satisfaction with the vacation is a very interesting variable, because, depending on the level of satisfaction or dissatisfaction, the degree of tourist loyalty to both the geographical area and the accommodation can be calculated. For Serra (2011, p. 122), after the vacation the tourist, through introspection, evaluates the experience and a feeling of satisfaction or dissatisfaction is created. As a result, a post-trip attitude is generated. This modifies several factors, such as the tourist's perception of the destination and attitude toward it, and these in turn influence the destination image for the tourist and his or her relatives and friends. The development of a more digitalized and responsible economy is also highlighted from the point of the view of the influence on other consumers, as explained by Moreno-Izquierdo et al. (2018), in which the collaboration between citizens and tourists is the frame of reference. Sevilla and Rodríguez (2019) emphasize the emotion caused by viewing the landscape during the journey and stay, which produces a satisfactory or unsatisfactory response to the tourist's expectations. Park et al. (2018) concluded that providing additional information before each visit can improve tourist satisfaction. Fernández-Herrero et al. (2018), state that tourist “autonomy improves overall satisfaction with the destination,” while Rojas-De-Gracia and Alarcón-Urbistondo (2019) explain the link between satisfaction and the decision-making process.

In studying tourist satisfaction, it is very important to perform multilevel analysis. This enables us to see the “big picture” of the factors affecting overall tourist satisfaction (Radojevic et al., 2017). The link between destination image and satisfaction has been widely researched. The study by Olague de la Cruz et al. (2017) focuses on the link between tourist motivation, destination image and satisfaction. They explain how motivations influence both cognitive and affective image, and both of this influence tourist satisfaction. For Rajesh (2013), destination image has a direct influence on both overall satisfaction and destination loyalty. Additionally, tourist satisfaction influences destination image—in other words, the new destination image a tourist generates after the vacation depends on the level of satisfaction. It's important to review the research by Martín et al. (2016), into the influence of destination image on satisfaction, and of satisfaction on loyalty. Herle (2018), Cruz et al. (2018), Machado et al. (2009), Huete and López (2020) and López-Sanz et al. (2021a) also researched this relationship. And we wish to highlight the study of Nysveen et al. (2018), who found a link between “green destination image” and tourist satisfaction. The expectancy disconfirmation theory will be used to explain the relationship between variables. This theory is very popular in consumer satisfaction research (Elkhani and Bakri, 2012; Kim et al., 2014). Positive disconformation happens when the final result is higher than initially expected, while negative disconformation happens when product performance and the final result is lower than expected at the beginning. Thus, we define the following hypothesis:

Hypothesis 4 (H4): “Destination image has a positive influence on overall trip satisfaction.”

Correia et al. (2013), explain that there is a relationship between the motivational “push” and “pull” variables and overall tourist satisfaction. Battour et al. (2012), who concluded that tourist motivation positively influences vacation satisfaction, should also be reviewed. For their part, Hidalgo-Fernández et al. (2019) also conclude in their study that there is a relationship between the motivations or interests of the tourist and satisfaction with the trip, turning this satisfaction into recommendation of the destination. This relationship is also found in their study Forteza et al. (2017) and He and Ming (2020).

The decision to choose the Spanish province of Soria was taken because of several factors. First, this is Spain's least populous province [a population of 88,658 in 2020 (Instituto Nacional de Estadística (INE), 2021)], and this area is suffering a worrying level of depopulation. And, on the other hand, it is a province with great potential from the touristic point of view, because it has a wide variety of natural and cultural resources. The province includes many very different areas: the highlands, with a special landscape and similar weather to the Scottish Highlands (hence its name); cities with an important cultural heritage, such as El Burgo de Osma and Soria itself; very interesting archeological areas including Numancia, La Dehesa's Roman Villa and the ancient village of Tiermes; and attractive natural sites such as La Laguna Negra, the Lobos River Canyon and the Fuentona sinkhole.

This province therefore can and must leverage the Rural Tourism boom in Spain and implement rural development based on the service sector, not only in terms of the increasing amount of accommodation available, but also through all the related activities. This includes promoting tourist routes, both cultural through the province's many heritage sites, and natural routes, that can in turn link with adventure and sports tourism. The province can also promote “experience-based tourism,” as explained by Mazarrasa (2016). This kind of tourism offers some activities which are relatively passive, such as visiting a winery to observe the steps in wine production. There are also, however, activities for which the tourist can actively participate in the experience.

The significance of this study lies in the fact that it can be a starting point for the right marketing actions to improve Rural Tourism in the area and prevent depopulation to the extent possible. To be successful, these actions must be supported by the national, local, provincial and regional authorities.

## Methods

### Survey Design

This research is based on a descriptive study using primary data from a questionnaire used on a representative sample of tourist over 18 years old who visited the province of Soria (Spain) and stayed in a rural tourism establishment. The primary selection of the different items of constructs was based on a review of the literature. Previously, the items had been carefully chosen, and before sending out the survey, preceding qualitative research was carried out through a focus group, which included five professors who are experts in tourism and consumer behavior. As a result of this qualitative research, the final questionnaire was achieved, consisting of four constructs with a total of 16 items: five for cognitive image (Baloglu and McCleary, 1999); two for affective image (Baloglu and McCleary, 1999); seven for tourist motivation (Crompton, 1979) and two for tourist satisfaction (Lee, 2009). In order to obtain data to analyze, 1,658 valid questionnaires were completed by adult tourists who stayed in a rural tourism establishment in the province, between January 2016 to January 2017, which implies a sampling error of ± 2.45% (with a confidence interval of 95.5% and p = q = 0.5) (see Table 1).

**Table 1:** Technical details of the study.

-Universe: tourists aged over 18 who stayed in a rural tourism establishment
-Place where interviews werw conducted La Laguna Negra Natural Park, Vinuesa, Calatañazor, Yanguas and Garray villages, The Lobos River Canyon
-Field work: From January 2016 to January 2017
-Geographical scope: provincial (Spanish province of Soria)
-Sample size: 1,658 valid questionnaires
-Sample design: structured questionnaire, anonymous. Personal interview
-Sampling error = 2.45% with 95.5% confidence level and p=q=50%

The data was collected through personal surveys. All items of the questionnaire used the same 4-point Likert-type scale, where 4 = a lot and 1 = little bit, except affective image and satisfaction items, where the scale was a 5-point Likert scale where 5 = strongly agree and 1 = strongly disagree (see Table 2).

**Table 2:** Scales of the model's constructors.

**Construct and items**	**Sources of adoption**
**Cognitive image**	
I identify the province of Soria with ease of playing sports	
I identify the province of Soria with favorable climate	
I identify the province of Soria with adventure opportunity	Baloglu and McCleary (1999)
I identify the province of Soria with aimed at both adults and families	
I identify the province of Soria with good road communication networks in the area	
**Affective image**	
I identify the province of Soria with relaxation	Baloglu and McCleary (1999)
I identify the province of Soria with pleasant	
**Motivation**	
Exploration and evaluation of self	
To be in contact with nature	
To participate in sports	Crompton (1979)
To attend cultural or religious events	
To recreate by gone days with the comfort of modern life	
Enhancement of kinship relationships	
Facilitation of social interaction	
**Satisfaction**	
You can value, in terms of satisfaction, your visit to the province of Soria	Lee (2009)
Considering your expectations, as you would value the experience in the province	

A pretest of this questionnaire was performed on 50 people who had visited the province and stayed in a rural tourism establishment, to evaluate if the scales were well-constructed and the multiple questions on the questionnaire were understood. After checking that everything was correct, the data were collected personally in the tourist areas of Soria province.

### Sample Size and Composition

The total sample consisted of 1,658 valid questionnaires of visitor over the age of 18 who were staying in a rural tourism establishment in the province of Soria (see Table 3).

**Table 3:** Sample information.

**Age**	**%**	**Education level**	**%**
18–35	20.08	Primary	23.52
36–45	46.14	Secondary	33.23
46+	33.77	University	43.24
**Marital status**	**%**	**Occupation**	**%**
Single	21.59	Employed	69.12
Married	41.38	Unemployed	29.55
Separated/divorced	10.37	Other	1.32
Living as a couple	26.54		
Widow(er)	0.12		
Gender	%		
Male	51.75		
Female	48.25		

The purpose of analyzing the information collected is to transform it into relevant information that assists the decision-making process. Several statistical techniques were applied to the data collected in the research, including Principal Component Analysis (PCA), and a model was prepared using Partial Least Squares Structural Equation Modeling (PLS-SEM). The programs used were IBM SPSS Statistic, DYANE 4 (Santesmases, 2009) and SmartPLS 3.2.28 (Ringle et al., 2015). Hair et al. (2011; p. 144) recommend selecting PLS-SEM if the research is exploratory or an extension of an existing structural theory. Hair et al. (2014) also recommend using PLS-SEM when formatively measured constructs are part of the structural model, the structural model is complex (many constructs and many indicators) and the data are non-normally distributed. It is possible to find these issues in this model, including a very complex structural model that was presented in the first moment.

## Results

### Factor Analysis of Variables

To facilitate the analysis of some of the variables studied, a factor analysis was performed. The chosen technique was Principal Component Analysis (PCA), a factor analysis technique that reveals the underlying dimensions or factors in the relationships between the values of the variables analyzed (Harman, 1976). It is a statistical method used to summarize and structure the information of a data matrix to reduce the number of variables (Lozares and López, 1991). The aim of this method is to reduce the number of dimensions by obtaining linear combinations with maximum variance that are uncorrelated to the original variables (Aguilera et al., 1996). In this study, we have used this technique to reduce the number of variables for the destination image and motivations constructs, due to their high number of variables. After our analysis, the cognitive destination image, which started with 31 variables, had just five factors, “variety of natural attractions vs. situational elements,” “cultural interest,” “entertainment and luxury,” “restful and attractive environment,” and “attractive accommodation.” Regarding affective image, we started with four variables that were reduced to two factors, “internal affective image” and “external affective image.” Finally, for motivations, the initial 23 variables were reduced to five factors, “cultural and natural,” “social,” “personal,” “novelty,” and “escape.”

Having retained the relevant information in the factors, as mentioned above, this research aims to find possible links between motivations, rural tourism destination image and tourist satisfaction for Spain's Soria province. The research focuses on studying the direct and indirect relationships between the variables. To analyze the cause-effect relationships between latent constructs (Hair et al., 2011) the Partial Least Squares (PLS) technique, which enables researchers to examine the structural component of a model (Gefen et al., 2000), was chosen. PLS-SEM has advantages over other SEM tools, such as LISREL, because PLS can be applied to explore the underlying theoretical model (Gefen et al., 2000). PLS-SEM doesn't require restrictive distributional assumptions about the data (Compeau et al., 1999), and the use of consistent PLS (PLSc) corrects the behavior of relationship coefficients between latent variables in reflective constructs. If, as in our study, the results are very similar, it is not necessary to apply this algorithm, but the deviations of the model's path coefficients are minimized (Dijkstra and Henseler, 2012).

### Behavior Model

The research studies the links between seven measured variables (Figure 1). This required a selection to be made.

For tourist motivations, we started with five factors (Table 4), but only cultural and natural motivations, and social motivations, have a loading of at least 0.3. The other ones (personal, novelty and escape), don't reach the required level. The valid items of every motivation factor are shown in Table 4.

**Table 4:** Rotated components matrix (Varimax method).

**Tourist motivations**	**Factor 1**	**Factor 2**	**Mean**	**Standard deviation**
Mot01 Exploration and evaluation of self	0.7568		2.76	1.00
Mot02 To be in contact with nature	0.8257		2.60	1.10
Mot03 To participate in sports	0.8680		2.55	1.12
Mot04 To attend cultural or religious events	0.7237		1.99	0.73
Mot05 To recreate bygone days with the comfort of modern life	0.7509		2.72	1.00
Cronbach's Alpha	0.721			
Mot06 Enhancement of kinship relationships		0.6585	1.55	0.75
Mot07 Facilitation of social interaction		0.6821	1.77	0.68
Cronbach's Alpha		0.675		
**Destination image**	**Factor 1**	**Factor 2**	**Mean**	**Standard deviation**
ImC01 Opportunity to participate in sport	0.7882		2.93	1.06
ImC02 Favorable climate	0.8992		2.66	1.22
ImC03 Opportunity for adventure	0.5539		3.37	0.76
ImC04 Suitable for both adults and families	0.7237		3.04	0.73
ImC05 Good transport network in the area	0.7515		2.77	0.99
Cronbach's Alpha	0.852			
ImA01 Relaxation		0.7274	4.25	0.72
ImA02 Pleasant		0.7467	4.26	0.71
Cronbach's Alpha		0.675		
**Tourist satisfaction**	**Factor 1**	**Mean**	**Standard deviation**	
Satisim Considering your expectations, how do you evaluate your experience in the province?	0.9644	4.45	0.76	
Satisfac Evaluate, in terms of satisfaction, your trip to Soria province	0.9644	4.71	0.48	
Cronbach's Alpha	0.884			
Bartlett test	6997.10			

The destination image variable may be composed of the factors of the cognitive and affective images (Table 4). Of the seven factors obtained for the cognitive and affective images, only variety of natural attractions vs. situational elements image, for cognitive image, and internal affective image, for affective image, have a loading of at least 0.3 or more on their constructs, resulting in seven valid items (Table 2). To measure the satisfaction, tourist perception was used, based on the abovementioned theoretical discussion, with two items: destination satisfaction and satisfaction in terms of expectations.

Using all these factors, we presented a theoretical model, as seen in Figure 1. The abovementioned link, between motivation, image and satisfaction is shown, as well as the factors that affect them most strongly.

The questionnaire was designed to measure seven different latent constructs: motivation (a second order construct with two dimensions), destination image (a second order construct with two dimensions) and satisfaction. The factor analysis was run to separately validate the measurement of those constructs. The Varimax rotation was used to assist in understanding the initial factor model. The factorial loads (see Table 4) provide evidence for the factorial validation of the scales.

The PLS measurement model is evaluated in terms of the inter-construct correlations, the correlations between items, Cronbach's Alpha, the reliability and the average variance extracted for every construct (AVE). In this case, the seven latent variables (two of which are second order constructs) are made up of scales with reflective items. The reliability, internal consistency and discriminant validity of every component in this study are assessed below.

The reliability assessment examines how each item is linked to the latent construct (Table 4). In this respect, the most generally accepted and widely used empirical rule is the one proposed by Carmines and Zeller (1979), who state that, to accept an indicator as part of a construct, it must have a loading ≥0.707. In this case, only one of the 16 indicators used (Table 2) doesn't reach this acceptable reliability level. However, as Chin (1998) and Barclay et al. (1995) explain, a loading of at least 0.5 can be acceptable if other indicators that measure the construct have higher assessed reliability. Furthermore, Falk and Miller (1992) propose a loading of 0.55—in other words, 30% of the variance of the manifest variable is related to the construct. The loading-−0.64—that didn't exceed the first condition did exceed these latter proposed levels and has a higher loading in its construct than in any other. These results strongly support the reliability of the reflective measurements (see Table 5).

**Table 5:** Model cross loading.

	**Cultural and natural motivations**	**Affective image**	**Cognitive image**	**Satisfaction**	**Social motivations**
ImA1	0.3241	**0.7733**	0.3217	0.207	0.2132
ImA2	0.3394	**0.7594**	0.3079	0.2358	0.2145
ImC2	0.8538	0.4017	**0.9095**	0.5436	0.5289
ImC3	0.4799	0.2433	**0.6432**	0.3017	0.2654
ImC4	0.6561	0.2989	**0.7688**	0.4086	0.4149
ImC5	0.6776	0.3118	**0.7907**	0.4508	0.4218
ImC1	0.7297	0.3595	**0.8437**	0.4442	0.4299
Mot2	**0.856**	0.4022	0.7461	0.4976	0.5405
Mot3	**0.8914**	0.3898	0.8179	0.5194	0.5314
Mot4	**0.7836**	0.3012	0.648	0.4285	0.4092
Mot5	**0.7956**	0.3366	0.6683	0.4325	0.3967
Mot1	**0.8059**	0.3532	0.6882	0.4665	0.4418
Mot6	0.6198	0.2928	0.5812	0.3672	**0.9308**
Mot7	0.3206	0.1796	0.2948	0.1869	**0.8269**
Satis im	0.6057	0.2995	0.5833	**0.9467**	0.3475
Satisfac	0.2949	0.1663	0.2846	**0.7598**	0.1874

*Bold indicates the most important values in the different study variables*.

Finally, motivation and image are valued as second-order reflective constructs for a molar model (Chin, 2010). The above discussion provides a basis for supporting the quality of the measurements of the components of these second order variables. The loadings of the dimensions of these constructs are also of interest. The statistics for all the dimensions were as expected, except for affective image, whose loading as a second order variable of image is 0.587 and therefore doesn't reach the AVE level of 0.707, although it exceeds the value of 0.55 (see Table 6).

**Table 6:** Internal consistency and AVE.

**Construct**	**Composite reliability**	**AVE**	**rho_A**
Image	0.865	0.492	0.853
Affective image	0.740	0.587	0.298
Cognitive image	0.895	0.634	0.867
Motivation	0.905	0.581	0.889
Cultural and natural motivation	0.873	0.775	0.888
Social motivation	0.847	0.737	0.815
Satisfaction	0.916	0.685	0.912

With respect to internal consistency, two measurements are taken into consideration, Rho value (rho_A) and Composite Reliability (see Table 6). Nunnally and Bernstein (1994) suggests 0.7 as a level indicating “modest” reliability which is suitable for the early stages of research, and a stricter one of 0.8 for basic research. As shown in Table 6, both indicators exceed the 0.8 value (except affective image, for which composite reliability is > 0.7 and Rho value is under 0.3).

Absolute fit indices determine how well a priori model fits the sample data (McDonald and Ho, 2002). These measures provide the most fundamental indication of how well the proposed theory fits the data. Included in this category is the Standardized Root Mean Square Residual (SRMR). The SRMR is an absolute measure of fit and is defined as the standardized difference between the observed correlation and the predicted correlation. Thus, it allows assessing the average magnitude of the discrepancies between observed and expected correlations as an absolute measure of (model) fit criterion. A value < 0.10 or of 0.08 are considered a good fit (Hu and Bentler, 1999). For this research model SRMR is 0.069 (below 0.08). Incremental fit indices, also known as comparative (Miles and Shevlin, 2007) or relative fit indices (McDonald and Ho, 2002), are a group of indices that do not use the chi-square in its raw form but compare the chi-square value to a baseline model. One of these indices is the Normed Fit Index (NFI). This statistic assesses the model by comparing the chi-square value of the model to the chi-square of the null model and values > 0.95 are recommended (Hu and Bentler, 1999) for a good fit. After the analysis it was found a NFI of 0.987 indicating a good fit.

The discriminant validity is obtained in two ways. First, the Average Variance Extracted (AVE) is examined, which indicates the amount of variance captured by the construct in relation to the variance due to measurement error. The value must exceed 0.50 (Fornell and Lacker, 1981). As shown in Table 6, all the AVE values exceed that value, except for image construct, which is close to it (0.492). Secondly, the square root of AVE (in the diagonal of **Table 10**) is compared to the other constructs (below the diagonal in Table 7). These statistics suggest that every construct is stronger in its own measurement than in the measurements of other constructs.

**Table 7:** Correlation and square root of AVE for first order latent variables.

	**Cultural and natural motivation**	**Social motivation**	**Cognitive image**	**Affective image**	**Satisfaction**
Cultural and natural motivation	**0.828**				
Social motivation	0.564	**0.880**			
Cognitive image	0.765	0.526	**0.796**		
Affective image	0.433	0.279	0.411	**0.766**	
Satisfaction	0.568	0.333	0.547	0.289	**0.858**

*Bold indicates the most important values in the different study variables*.

Collectively, these results support the quality of the measurements. Specifically, the statistics suggest that the components of our measurements are reliable, internally consistent and they have discriminant validity.

### Results of SEM

A PLS estimated model allows us to establish the variance of the explained endogenous variables by the constructs that predict them. Falk and Miller (1992) suggest that the explained variance of the endogenous variables (*R*^2^) should be ≥0.1. Related to this model, the indexes (see Table 8) explain the large variance of the second order variables, because the *R*^2^ values of the dimensions (both image and motivation) exceed 0.5 (except in the case of the affective image, which exceeds 0.3). The *R*^2^ value for satisfaction also exceeds 0.3. Stone-Geisser's *Q*^2^ value must exceed 0, and this suggests a predictive relevance related to the endogenous construct model (Chin, 1998). In this case, all the variables exceed that value (the lowest is satisfaction with a value of 0.2).

**Table 8:** R square and stone-geisser.

**Construct**	***R^**2**^***	***Q^**2**^***
Image	0.727	0.336
Affective image	0.345	0.196
Cognitive image	0.959	0.572
Cultural and natural motivation	0.941	0.606
Social motivation	0.559	0.403
Satisfaction	0.304	0.200

**Figure 2:**
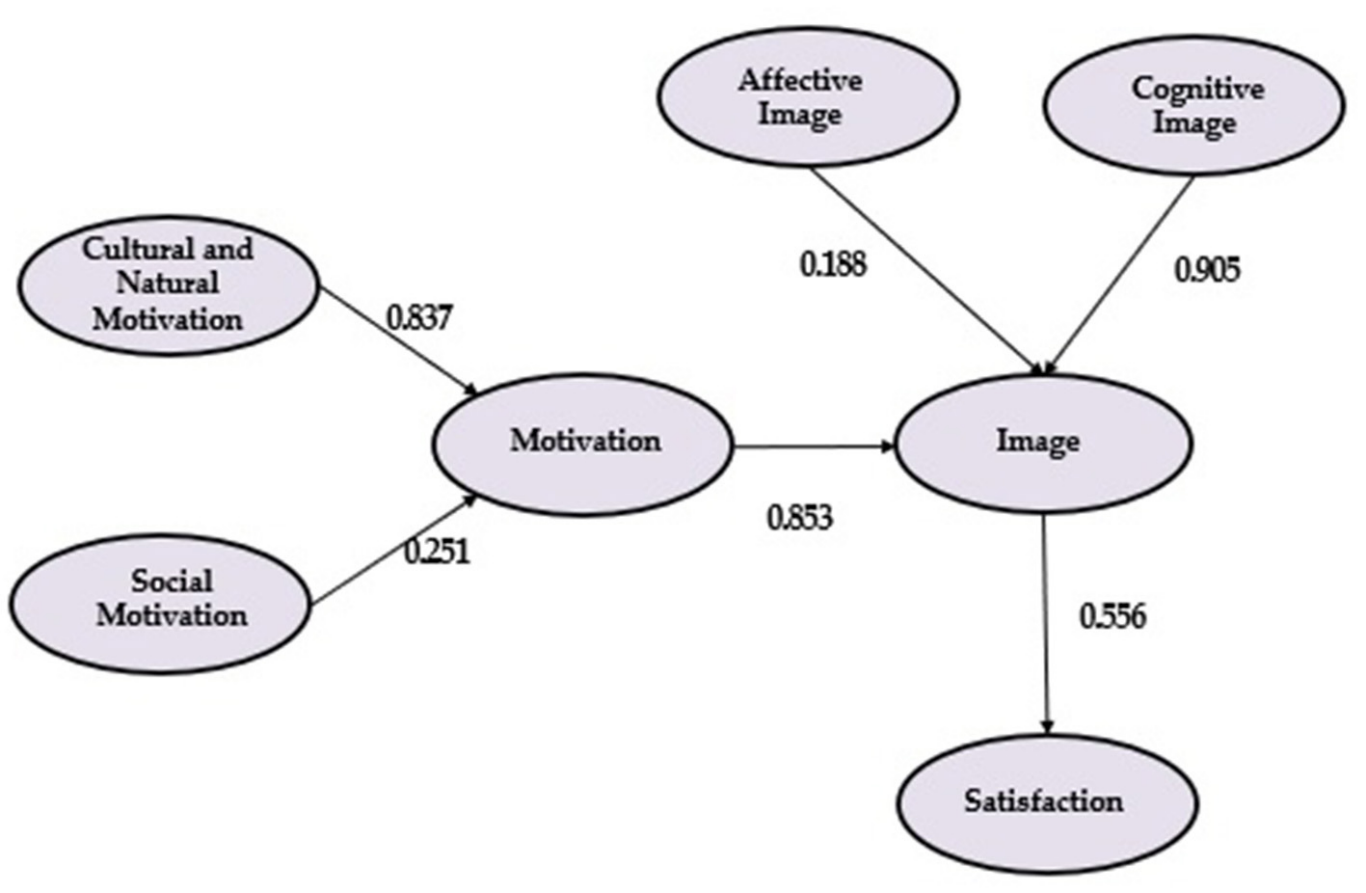
Results.

To obtain indications of external validity, image and tourist satisfaction need to be significantly linked with motivation, as the theory explains (Bagozzi, 1994). Based on this literature, a model in which motivation is a precedent and has a positive relationship with destination image was estimated, and this is also a precedent of satisfaction (see Figure 2).

Table 9 shows that the path coefficients are significant (*p* < 0.001) since there aren't any non-significant coefficients. The significance of the coefficients was estimated using PLS bootstrapping with 500 samples, an appropriate quantity to obtain reasonable estimations of standard error (Chin, 2010).

**Table 9:** Significance of the coefficients.

	**Coeficients**	**t-statistic (|O/STDEV|)**	***p*-values**
Image -> satisfaction	0.556	39.165	0.000
Affective Image -> image	0.188	22.895	0.000
Cognitive image -> image	0.905	123.156	0.000
Cultural and natural motivation -> motivation	0.837	135.003	0.000
Social motivation -> motivation	0.251	47.113	0.000
Social motivation -> image	0.085	3.718	0.000

And since one of our hypotheses focuses on the indirect effect of the motivation with satisfaction variable, we can observe the existing relationship (0.47) through the results of Table 10.

**Table 10:** Direct and indirect effects of the coefficients.

	**Direct effects**	**Indirect effects**
Motivation → image	**0.853**	
Image → satisfaction	**0.551**	
Motivation → satisfaction		0.470

*Bold indicates the most important values in the different study variables*.

In summary, in the model there is a direct and strong link between motivation and destination image (0.853). Motivation thus seems to be an important element influencing destination image. We have therefore proven that our hypothesis 2, “Tourist motivation significantly positive influences destination image formation” is correct (see Table 11).

**Table 11:** Summary of hypothesis verification.

**Hypothesis**	**Content**	**Verification**
H1.1	Cultural and natural motivation is the main dimension of the tourist motivation	Supported
H1.2	Social motivation has an important relevance in the formation of tourist motivation	Rejected
H2	Tourist motivation significantly positive influences destination image formation	Supported
H3.1	Affective destination image has a positive influence on destination image formation	Supported
H3.2	Cognitive destination image has a positive influence on destination image formation	Supported
H4	Destination image has a positive influence on overall trip satisfaction	Supported

Regarding the hypotheses 1.1 and 1.2, “Cultural and natural motivation is the main dimension of the tourist motivation” and “Social motivation has an important relevance in the formation of tourist motivation,” the dimension of cultural and natural motivation is the one that reflects motivation (0.837) better than the other dimension of motivation, social motivation (0.251). It is possible that the type of motivation that is most influential will vary depending on the characteristics of the destination. In this case, motivations related to nature and culture are the most significant (see Table 11).

It is the cognitive image dimension that best reflects destination image (0.905) and there are some problems in considering the affective image to be a good reflection of destination image. The hypothesis 3.1, “Affective destination image has a positive influence on destination image formation” is therefore incorrect, while hypothesis 3.2, “Cognitive destination image has a positive influence on destination image formation” is correct. There is also a positive and direct link between destination image and satisfaction (0.556), and as a result we can accept the hypothesis 4, “Destination image has a positive influence on overall trip satisfaction” (see Table 11).

On the other hand, and indirectly, a relatively important link (0.470) between motivation and satisfaction has been found (see Table 10), especially if we consider the current difficulty in influencing satisfaction. This is a consequence of a strong link, which is direct and positive, between motivation and destination image. This relationship between tourist motivations and satisfaction was studied by Correia et al. (2013), explained that there is a relationship between the motivational “push” and “pull” variables and overall tourist satisfaction. Battour et al. (2012), who concluded that tourist motivation positively influences vacation satisfaction, should also be reviewed. For their part, Hidalgo-Fernández et al. (2019) also conclude in their study that there is a relationship between the motivations or interests of the tourist and satisfaction with the trip, turning this satisfaction into recommendation of the destination. This relationship is also found in their study Forteza et al. (2017) and He and Ming (2020). Thus, we can check that motivation seems to be an important element in influencing both destination image and satisfaction, which has significant entrepreneurial consequences.

## Discussion and Conclusions

### Theorical Discussions

This study aims to analyze how rural tourism, in line with the Sustainable Development Goal number 8 of the UNWTO (World Tourism Organization (UNWTO), 2021), can serve to sustainably develop the most depopulated rural areas (Marzo-Navarro, 2017). We must focus on the social and economic sustainability of this type of tourism, which should translate into improving the quality of life of the indigenous population of the area (Pérez de la Heras, 2004), and culturally and socially enriching the local population (Rytkönen and Tunón, 2020). The social well-being of local economies is linked to tourism in those areas (Tasci, 2017) and increases the sustainability of the local population.

The analysis of rural tourism has been carried out through the relationship that exists between the motivations that move the tourist (Dann, 1977; Wong et al., 2018), which is one of the most important variables for decision-making in tourism (Prebensen et al., 2010; Polo et al., 2016; Line et al., 2018; González and Vallejo, 2021); the image of the tourist destination, a key factor when tourists are choosing their destinations, and crucial when planning a trip (Marine-Roig and Ferrer-Rosell, 2018); and satisfaction with the trip, a relationship studied by Forteza et al. (2017), Hidalgo-Fernández et al. (2019) and He and Ming (2020). This relationship has served to study the behavior of rural tourists related to sustainable development goals, especially goal number 8 “decent work and economic growth.”

From an academic point of view, the proposed Structural Equation Model could be used in many studies researching the links between the three variables studied (tourist motivation, destination image and trip satisfaction), because its reliability and predictive capacity have been proven, as shown by the results obtained. It is not only useful for research into rural tourism, but also for general tourism research, as well as for research into other kinds of rural tourism that have recently become popular, such as adventure tourism, sport tourism, cultural tourism and, in countries with a traditional wine industry, wine tourism.

Summary, we have demonstrated the importance of these three variables in the study of the rural tourism behavior and, thanks to this study, real and effective measures can be taken for the sustainable development of the rural area and thus be able to meet the objective number 8 of the UN Sustainable Development Goals.

### Managerial Discussions

From a managerial point of view, this research can assist all those authorities that influence rural tourism policies in Spain's Soria province and the rest of Spain, when making policies to promote this kind of tourism, specially promoting the cognitive image that each of us have of a tourist area. We have seen the importance to these rural areas, the country's most depopulated, of tourism (Flores and Barroso, 2012) as a complement to their more traditional activities (principally agriculture and livestock). Depopulation in these areas is a critical problem (del Romero, 2018), since in some places, including some that offer rural, cultural, and natural attractions, the population has almost completely disappeared. This also leads to a loss of heritage for the province and for the country in general.

The results obtained demonstrate the importance of studying the variables used, especially the image of the tourist destination (Beerli et al., 2003), for the promotion of the tourist area. This promotion seems very important, as explained by Baloglu and McCleary (1999) and Zhang et al. (2018). And as we have verified, this image is formed especially as a result of the knowledge we obtain about the destination (Sanz, 2008), much more than from the feelings that the destination causes in us.

It is also important, as Prebensen et al. (2010), Polo et al. (2016), Line et al. (2018), and González and Vallejo (2021) explained, to analyze the motivations that drive tourists. Sancho and Álvarez (2010) point out the importance of motivation in the decision-making process. Therefore, the different administrations involved in tourism policies, as well as the owners of rural establishments, should consider the different motivations that influence decision-making (Wong et al., 2018), as well as the formation of the community destination image (Mayo and Jarvis, 1981; Michie, 1986; Gong and Sun Tung, 2017). In addition, due to the indirect but strong link between tourist motivations and satisfaction with the trip (Fernández-Herrero et al., 2018), the need to cover these motivations must be considered, especially cultural, natural and social motivations (Penelas-Leguía et al., 2019), so that the tourist has a satisfactory trip, which will positively influence loyalty with the destination (López-Sanz et al., 2021b) and will have an impact on better business results for tourist establishments of the area (Moliner et al., 2009).

From the point of view of the Sustainable Development Goals, especially Goal 8, “Decent Work and Economic Growth,” the development of rural tourism can directly help to achieve this SDG (Alcivar, 2020), as well as to avoid depopulation that threatens these regions of Spain so much (Maroto and Pinos, 2020), promoting quality employment and avoiding exodus to the city and to other richer areas.

### Limitations and Future Research

The main limitation of this study is that we have focused on a Spanish province. It would be convenient to apply this methodology to a complete study, focusing on the Autonomous Community of Castilla y León, to which Soria belongs, or even the entire Spanish state. A comparative study could also be made with other provinces with similar levels of depopulation in Spain, to compare both the strategies that are carried out in each of them, as well as the differences in the motivations that move tourists to those other provinces like the image that each one projects.

Another future line of research would be to extend the study to other different motivational factors, not only natural and cultural and social, to obtain other conclusions about tourist behavior. In addition, due to the discovery of the strong indirect effect that tourist motivations have on satisfaction, the study could be extended toward loyalty with the destination, and check if this indirect effect also applies between the tourist motivations and loyalty with the destination.

Finally, a similar study could be carried out by directing the questionnaire to tourists who focus on nature tourism, to discover any differences between them and rural tourists.

## Conclusions

Therefore, if we look at in the principal and secondary objectives, the proposed model (Figure 2) below, shows the direct link between the motivations that drive a tourist and his or her perceived destination image, as well as between image and overall tourist satisfaction with the trip. A link between motivations and satisfaction has been demonstrated, although it is indirect. These relationships demonstrate the importance of these three variables in the rural tourist behavior.

This study is important to be able to make decisions, especially from the point of view of local, regional and national tourism policies, to promote sustainable rural development and economic growth in the area, promoting job creation, to meet the Goal number 8 of Sustainable Development. With this economic development, a sustainable social development is directly achieved that is one of the pillars for the eradication of inequalities and poverty in rural areas.

## Data Availability Statement

The raw data supporting the conclusions of this article will be made available by the authors, without undue reservation.

## Author Contributions

All authors contributed to conception and design of the study, organized the database, performed the statistical analysis, wrote the first draft of the manuscript, wrote all the sections of the manuscript, contributed to manuscript revision, read, and approved the submitted version.

## Conflict of Interest

The authors declare that the research was conducted in the absence of any commercial or financial relationships that could be construed as a potential conflict of interest.

## Publisher's Note

All claims expressed in this article are solely those of the authors and do not necessarily represent those of their affiliated organizations, or those of the publisher, the editors and the reviewers. Any product that may be evaluated in this article, or claim that may be made by its manufacturer, is not guaranteed or endorsed by the publisher.
